# Circular RNA hsa_circ_0003141 promotes tumorigenesis of hepatocellular carcinoma via a miR-1827/UBAP2 axis

**DOI:** 10.18632/aging.103244

**Published:** 2020-05-28

**Authors:** Yong Wang, Rongfen Gao, Jinpeng Li, Shaotao Tang, Shuai Li, Qiangsong Tong, Yongzhong Mao

**Affiliations:** 1 Department of Pediatric Surgery, Union Hospital, Tongji Medical College, Huazhong University of Science and Technology, Wuhan 430022, Hubei, P.R. China; 2 Department of Rheumatology and Immunology, Tongji Hospital, Tongji Medical College, Huazhong University of Science and Technology, Wuhan 430030, Hubei, P.R. China; 3 Department of Thyroid and Breast Surgery, Wuhan University Zhongnan Hospital, Wuhan 430071, Hubei, P.R. China

**Keywords:** hepatocellular carcinoma, hsa_circ_0003141, microRNA-1827, UBAP2, tumorigenesis

## Abstract

Circular RNAs (circRNAs) play an important role in the tumorigenesis of hepatocellular carcinoma (HCC), but their specific functions in HCC remain largely unknown. Using bioinformatics analysis, we have found that the expression of circRNA hsa_circ_0003141 is significantly increased in HCC tissues. Ubiquitin-associated protein 2 (UBAP2) is the parent gene for hsa_circ_0003141, and its high expression correlates with poor overall survival rates in HCC patients. In addition, our results show that miR-1827 is a binding target of hsa_circ_0003141, and indicate that hsa_circ_0003141 regulates UBAP2 expression by sponging miR-1827 in HCC cells. Downregulation of hsa_circ_0003141 suppresses UBAP2 expression, induces apoptosis, and inhibits proliferation and invasion by HCC Huh-7 cells. Importantly, downregulation of hsa_circ_0003141 inhibits tumorigenesis in a xenograft mouse model of HCC. Together, our results indicate that hsa_circ_0003141 functions as an oncogene in HCC cells, and suggest that the hsa_circ_0003141/miR-1827/UBAP2 axis might represent a novel therapeutic option for the treatment of HCC.

## INTRODUCTION

Hepatocellular carcinoma (HCC) accounts for approximately 90% of primary liver cancer, and is the third leading cause of cancer-related deaths in the world [1, 2]. One of the major risk factors for HCC is chronic liver infection caused by hepatitis B or C virus (HBV or HCV) [3]. Several treatment approaches are available for HCC, such as liver transplantation, chemoradiotherapy, and surgical resection [4]. However, the five-year overall survival rates of patients with HCC remain low, largely because of metastasis and recurrence [5, 6]. To improve diagnosis and prognosis of patients with HCC, it is critical to identify novel HCC biomarkers.

Circular RNAs (circRNAs) are non-coding RNAs that exist mainly in the cytoplasm [7]. They lack 5′-3′ ends and polyadenylated tail, and form covalently closed loops [8]. CircRNAs are more stable than linear RNAs because circRNAs are less susceptible to degradation by RNase R [9]. Many circRNAs have important biological functions and regulate behavior of tumor cells, including apoptosis, migration, and invasion [10, 11]; they have been also implicated in the carcinogenesis and progression of HCC [12]. CircRNAs regulate target mRNAs by acting as miRNA sponges [13].

MicroRNAs (miRNAs) are a class of non-coding RNAs that regulate expression of their target genes at the post-transcriptional level [14]. MiRNAs can function as oncogenes or tumor suppressors in cancer cells including HCC [15, 16], by regulating apoptosis, migration, invasion, and differentiation of tumor cells [17].

In the present study, we analyzed two GEO datasets to identify differentially expressed circRNAs (DEcircRNAs) between HCC tissues and matched normal tissues. We found that the circRNA hsa_circ_0003141 is significantly increased in HCC tissues, and promotes HCC tumorigenesis.

## RESULTS

### Identification of DEcircRNAs in HCC

**Figure 1:**
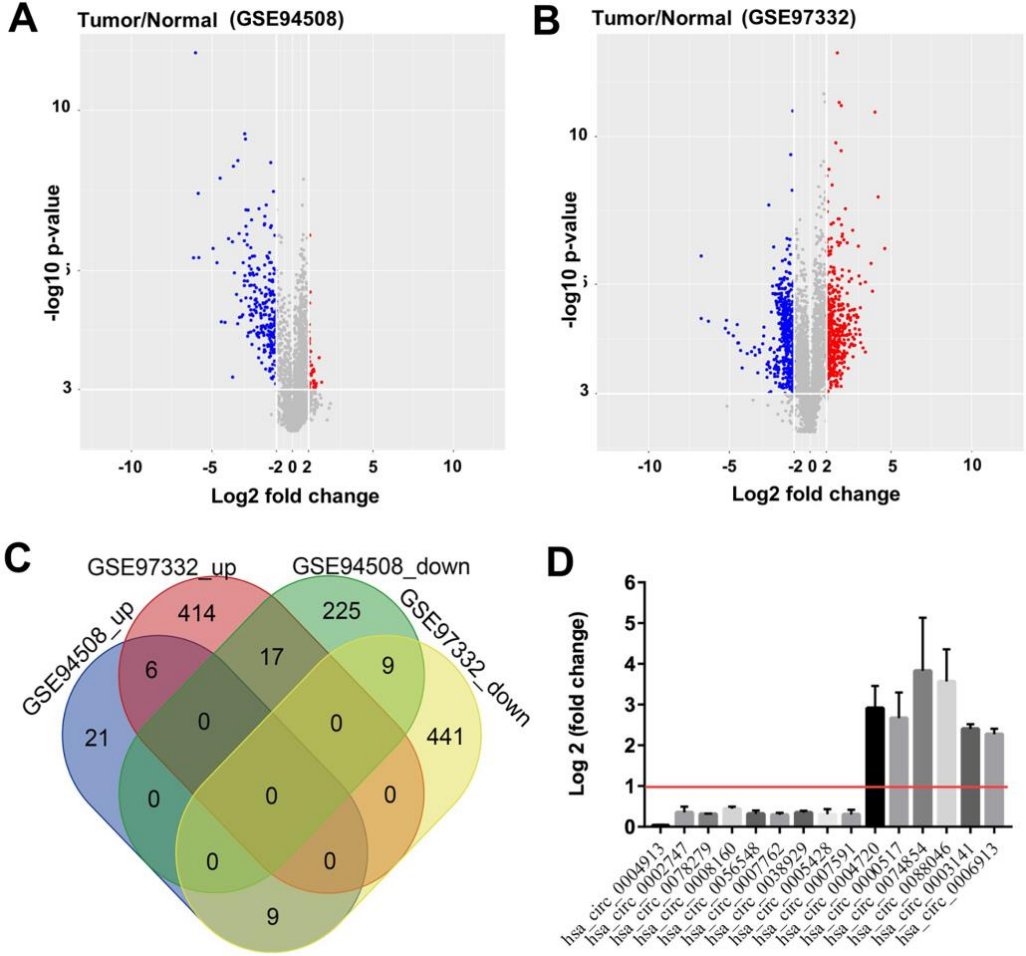
**Identification of DEcircRNAs in HCC.** Identification of DEcircRNAs in two GEO datasets using (**A**) Volcano plot of DEcircRNAs in GSE94508, and (**B**) Volcano plot of DEcircRNAs in GSE97332. High expression of DEcircRNAs is highlighted in blue, while low expression of DEcircRNAs is highlighted in red; P-value <0.001 (-log10 p-value > 3) and |log2 Fold Change| > 2 were set as thresholds. (**C**) DEcircRNAs from the two GEO datasets (GSE94508 and GSE97332) analyzed using Venn diagram. (**D**) 9 downregulated overlapping DEcircRNAs, and 6 upregulated overlapping DEcircRNAs were identified using R language.

To identify the differentially expressed circRNAs (DEcircRNAs) in HCC, we downloaded the GSE94508 and GSE97332 datasets from GEO, and analyzed the expression profiles of circRNAs by using the LIMMA package. A total of 287 DEcircRNAs were identified from the GSE94508 dataset; 251 were downregulated and 36 were upregulated. The distribution of DEcircRNAs is presented by volcano plot (Figure 1A). A total of 896 DEcircRNAs were identified from the GSE97332 dataset; 459 were downregulated and 437 were upregulated (Figure 1B). The intersect function identified 6 upregulated DEcircRNAs, and 9 downregulated DEcircRNAs from the two datasets using a Venn diagram (Figure 1C). The nine downregulated overlapping DEcircRNAs included hsa_circ_0004913, hsa_circ_0002747, hsa_circ_0078279, hsa_circ_0008160, hsa_circ_0056548, hsa_circ_0007762, hsa_circ_0038929, hsa_circ_0005428, and hsa_circ_0007591. The six upregulated overlapping DEcircRNAs included hsa_circ_0004720, hsa_circ_0000517, hsa_circ_0074854, hsa_circ_0088046, hsa_circ_0003141, and hsa_circ_0006913 (Figure 1D).

### GO and KEGG analysis of DEcircRNAs

**Figure 2:**
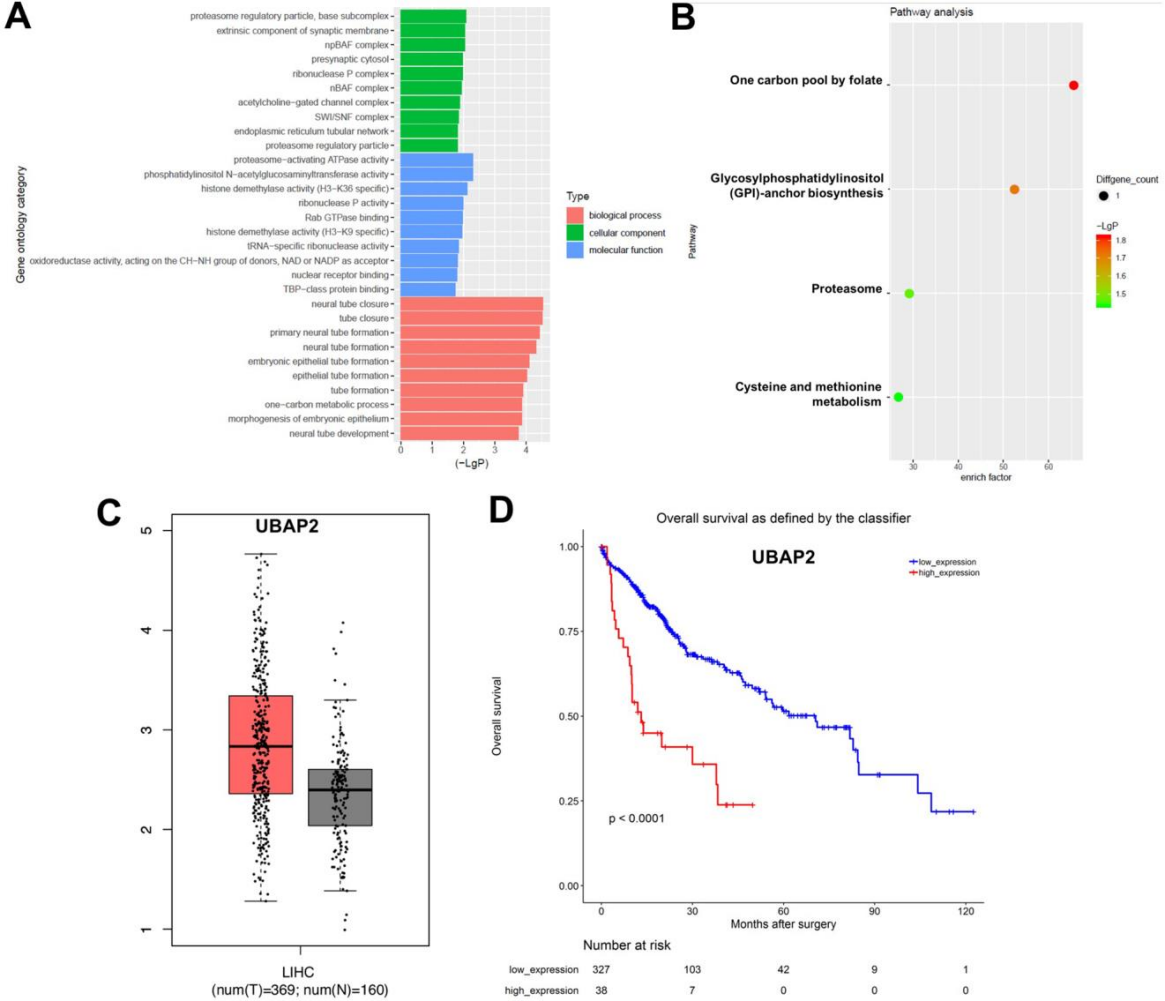
**GO and KEGG analysis of DEcircRNAs.** (**A**) Overlapping DEcircRNAs assessed by gene ontology (GO) analysis. (**B**) Overlapping DEcircRNAs assessed by Kyoto Encyclopedia of Genes and Genomes (KEGG) analysis. (**C**) Relative expression of UBAP2 expression in LIHC tissues (n = 369, T) and in normal tissues (n = 160, N) in TCGA dataset. (**D**) Survival analysis of the correlation between UBAP2 levels and survival rates in HCC patients using Kaplan–Meier Plotter online platform.

Next, the DEcircRNAs were analyzed using the gene ontology (GO) enrichment and KEGG pathway analyses. The GO results showed that DEcircRNAs were mainly enriched in “proteasome regulatory pathway”, “proteasome-activating ATPase activity”, and “one-carbon metabolic processes” (Figure 2A). The KEGG analysis showed enrichment in “one carbon pool by folate”, “glycosylphosphatidylinositol (GPI)-anchor biosynthesis”, “proteasome”, and “cysteine and methionine metabolism” (Figure 2B). In addition, the prognostic value of the overlapping circRNAs’ parent genes was analyzed using the Kaplan-Meier method from TCGA data. Ubiquitin associated protein 2 (UBAP2) is the parent gene of hsa_circ_0003141. As shown in Supplementary Figure 1A and 1B, the level of UBAP2 was much higher in Hep3B2.1-7 and Huh-7 cells than that in HepG2 and HCCLM3 cells, thus, Huh-7 and Hep3B2.1-7 cells were chose to conduct the following experiments. In addition, the analysis of the TCGA dataset revealed that UBAP2 levels in 369 liver hepatocellular carcinoma (LIHC) tissues were higher than that of in 160 normal tissues (Figure 2C). Moreover, high UBAP2 expression correlated with poor overall survival rates in patients with HCC (Figure 2D), indicating that increased hsa_circ_0003141 levels could predict worse survival outcomes in HCC patients.

### Downregulation of hsa_circ_0003141 inhibits proliferation of HCC cells

**Figure 3:**
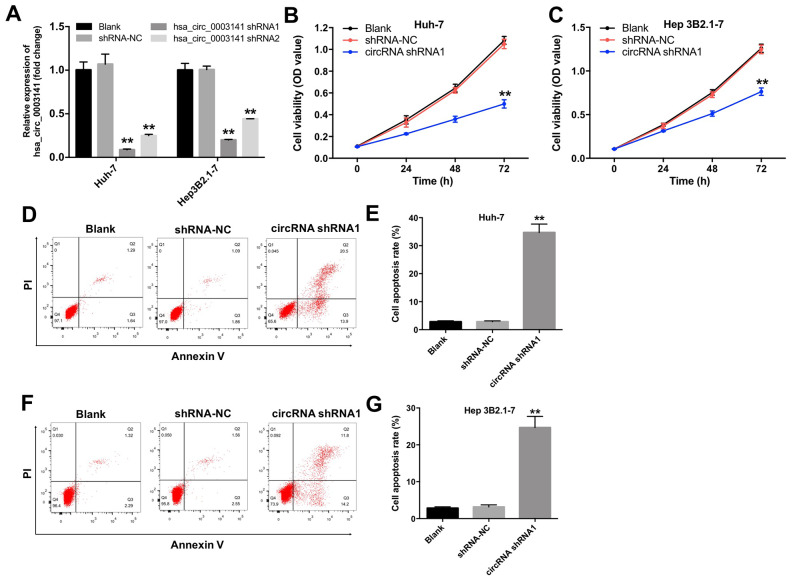
**Downregulation of hsa_circ_0003141 inhibits proliferation of HCC cells.** (**A**) The level of hsa_circ_0003141 in Huh-7 and Hep3B2.1-7 cells transfected with hsa_circ_0003141-shRNA1 or hsa_circ_0003141-shRNA2 analyzed by qRT-PCR. (**B**) Cell viability analyzed by CCK-8 assay in Huh-7 and (**C**) Hep3B2.1-7 cells transfected with hsa_circ_0003141-shRNA1 for 0, 24, 48 and 72 h. (**D**, **E**) Apoptosis measured by Annexin V and PI double staining in Huh-7 cells and (**F**, **G**) Hep3B2.1-7 cells transfected with hsa_circ_0003141-shRNA1 for 72 h. **P < 0.01, compared with the shRNA-NC group.

To investigate the role of hsa_circ_0003141 in HCC cells, we established HCC cell lines (Huh-7 and Hep3B2.1-7) with hsa_circ_0003141 stable knockdown. As shown in Figure 3A, the level of hsa_circ_0003141 was significantly downregulated in Huh-7 and Hep3B2.1-7 cells following transfection with hsa_circ_0003141-shRNA1. In addition, CCK-8 proliferation assay showed that downregulation of hsa_circ_0003141 suppressed proliferation of Huh-7 and Hep3B2.1-7 cells, compared with the shRNA-NC group (Figure 3B and 3C). Moreover, silencing of hsa_circ_0003141 greatly induced apoptosis of Huh-7 and Hep3B2.1-7 cells, compared with the shRNA-NC group (Figure 3D–3G). These data indicate that downregulation of hsa_circ_0003141 induces apoptosis and inhibits proliferation of HCC cells.

### Downregulation of hsa_circ_0003141 inhibits invasion of HCC cells

**Figure 4:**
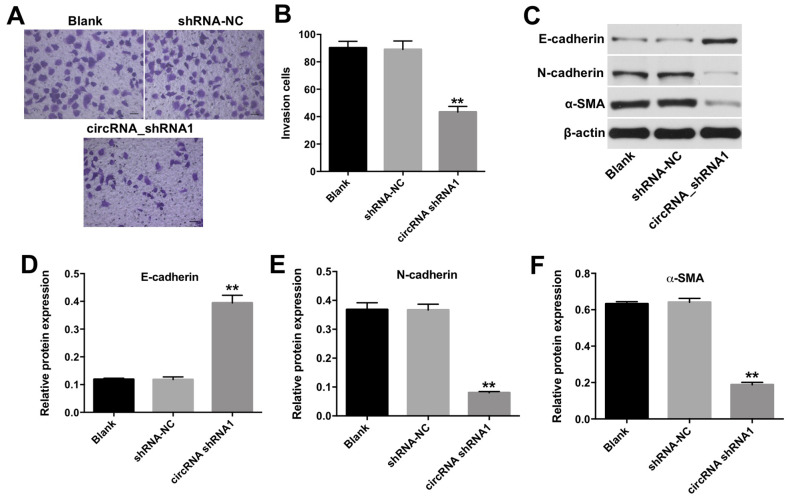
**Downregulation of hsa_circ_0003141 inhibits invasion of HCC cells.** (**A**, **B**) Huh-7 cells were transfected with hsa_circ_0003141-shRNA1 for 24 h. The invasion ability of Huh-7 cells was measured using transwell invasion assay. (**C**) Western analysis of E-cadherin, N-cadherin, and α-SMA levels in Huh-7 cells. (**D**–**F**) The relative expression of E-cadherin, N-cadherin and α-SMA in Huh-7 cells normalized to β-actin. **P < 0.01, compared with the shRNA-NC group.

To investigate the effect of hsa_circ_0003141 on invasion of HCC cells, transwell invasion assay was performed. As shown in Figure 4A and 4B, the invasion ability of Huh-7 cells was significantly reduced following transfection with hsa_circ_0003141-shRNA1. In addition, downregulation of hsa_circ_0003141 markedly increased the level of mesenchymal-to-epithelial transition (MET) marker E-cadherin, and decreased the levels of epithelial-mesenchymal transition (EMT) markers N-cadherin and α-SMA in Huh-7 cells (Figure 4C–4F). These results suggest that downregulation of hsa_circ_0003141 inhibits the invasion ability of HCC cells via inhibiting the EMT process.

### Hsa_circ_0003141 functions as a ceRNA of miR-1827 in HCC

**Figure 5:**
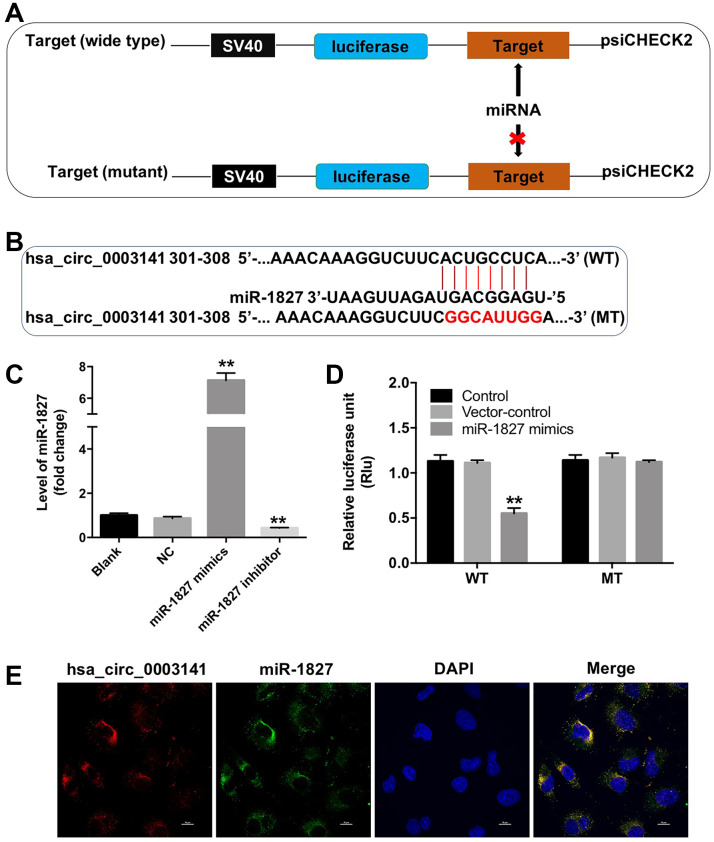
**Hsa_circ_0003141 functions as a ceRNA of miR-1827 in HCC.** (**A**, **B**) The 3'-UTR of hsa_circ_0003141 harbors miR-1827 cognate sites. (**C**) The level of miR-1827 in Huh-7 cells transfected with miR-1827 mimics or miR-1827 inhibitor analyzed by qRT-PCR. (**D**) Relative luciferase activity of reporter plasmids carrying WT- or MT-hsa_circ_0003141 3'-UTR in Huh-7 cells transfected with miR-1827, and analyzed using dual luciferase reporter assay. (**E**) Cellular localization of hsa_circ_0003141 and miR-1827 in Huh-7 cells was analyzed using FISH assay. **P < 0.01, compared with the vector-control group.

Circular RNA interactome (https://circinteractome.nia.nih.gov) was used to identify the miRNAs interacting with hsa_circ_0003141. As shown in Figure 5A and 5B, the potential binding sites for miR-1827 in hsa_circ_0003141 were predicted, indicating that miR-1827 is a potential binding target of hsa_circ_0003141. RT-qPCR assay showed that miR-1827 mimics significantly increased the level of miR-1827 in Huh-7 cells, and miR-1827 inhibitor significantly decreased the level of miR-1827, compared with NC group (Figure 5C). In addition, dual luciferase reporter assay showed that the luciferase activity was significantly lower in Huh-7 cells co-transfected with WT-hsa_circ_0003141 segment and miR-1827 mimics compared to vector-control group, while no significant difference was observed in Huh-7 cells co-transfected with MT-hsa_circ_0003141 segment and miR-1827 mimics (Figure 5D). Moreover, FISH assay revealed that hsa_circ_0003141 and miR-1827 are co-localized in the cytoplasm (Figure 5E). These data suggest that hsa_circ_0003141 may function as a competing endogenous RNA (ceRNA) of miR-1827 in HCC.

### UBAP2 is a direct binding target of miR-1827

**Figure 6:**
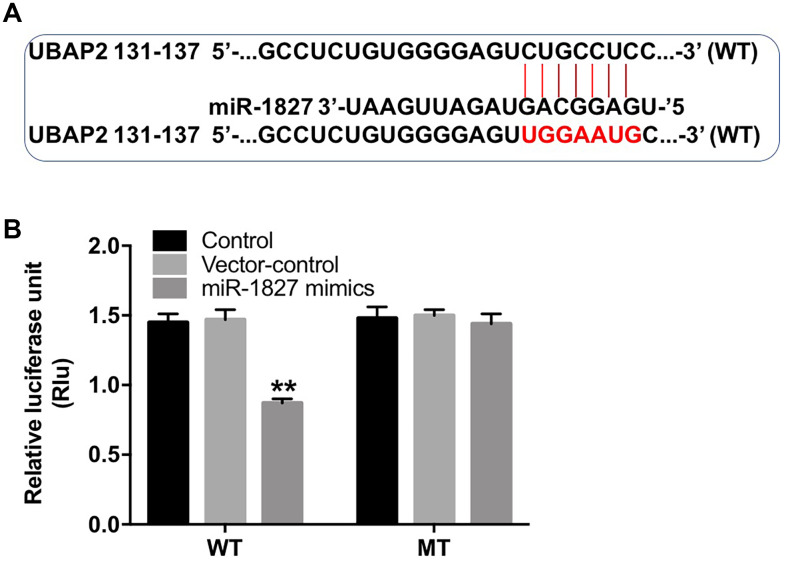
**UBAP2 is a direct binding target of miR-1827.** (**A**) The 3'-UTR of UBAP2 harbors miR-1827 cognate sites. (**B**) Relative luciferase activity of reporter plasmids carrying WT- or MT-UBAP2 3'-UTR in Huh-7 cells transfected with miR-1827 analyzed by dual luciferase reporter assay. **P < 0.01, compared with the vector-control group.

To identify the potential target of miR-1827, we used the TargetScan dataset (http://www.targetscan.org/vert_71/). The data analysis indicated that ubiquitin-associated protein 2 (UBAP2) might be a potential target of miR-1827 (Figure 6A). In addition, dual luciferase reporter assay showed that the luciferase activity was markedly lower in Huh-7 cells co-transfected with the WT-UBAP2 segment and miR-1827 mimics, compared to vector-control group (Figure 6B), indicating that UBAP2 is a direct binding target of miR-1827.

### Hsa_circ_0003141 functions as oncogene in HCC by sponging miR-1827, thus increasing UBAP2 expression

**Figure 7:**
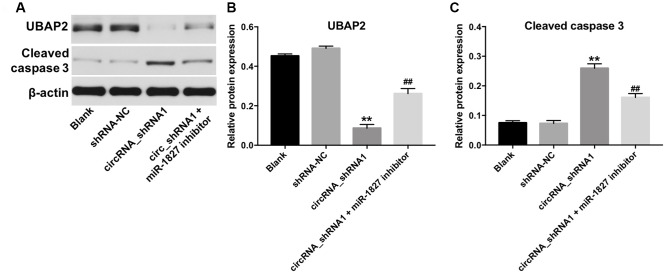
**Hsa_circ_0003141 functions as oncogene in Hun-7 cells by sponging miR-1827, and thus increasing UBAP2 expression.** (**A**) Western analysis of UBAP2 and cleaved caspase 3 in Huh-7 cells transfected with hsa_circ_0003141-shRNA1 with and without miR-1827 inhibitor. (**B**, **C**) The relative expression of UBAP2 and cleaved caspase 3 in Huh-7 cells normalized to β-actin. **P < 0.01, compared with the shRNA-NC group. ^##^P < 0.01, compared with the circRNA_shRNA1 group.

Next, we investigated the role of hsa_circ_0003141 and miR-1827 in the regulation of UBAP2 protein expression. RT-qPCR assay showed that miR-1827 mimics significantly increased the level of miR-1827 in Hep3B2.1-7 cells, and miR-1827 inhibitor significantly decreased the level of miR-1827, compared with NC group (Supplementary Figure 2A). As shown in Figure 7A**–**7C, and Supplementary Figure 2B–2D, downregulation of hsa_circ_0003141 significantly decreased the level of UBAP2, and increased the level of cleaved caspase 3 in Huh-7 and Hep3B2.1-7 cells, compared with shRNA-NC group. However, when Huh-7 or Hep3B2.1-7 cells were treated with hsa_circ_0003141-shRNA1 together with miR-1827 inhibitor, the effect of hsa_circ_0003141 on UBAP2 protein expression and cleaved caspase 3 was reversed by miR-1827 knockdown. These data indicate that hsa_circ_0003141 promotes HCC tumorigenesis by sponging miR-1827, thus increasing UBAP2 expression.

### Knockdown of hsa_circ_0003141 inhibits tumorigenesis in HCC xenografts *in vivo*

**Figure 8:**
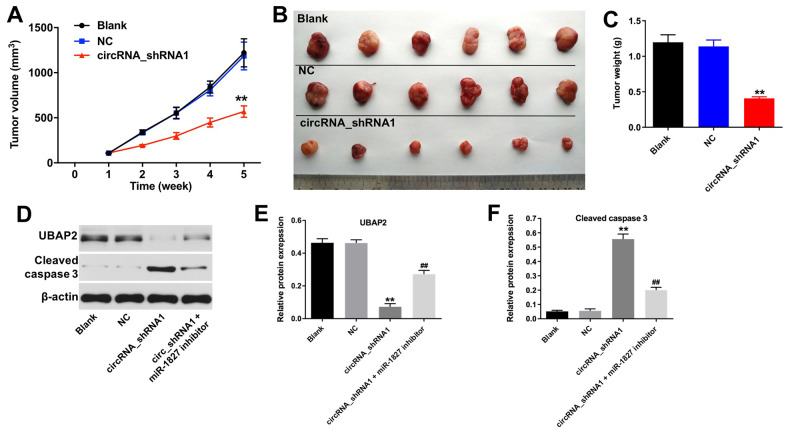
**Knockdown of hsa_circ_0003141 inhibits tumorigenesis of Huh-7 subcutaneous xenografts *in vivo*. Mice were s.c. implanted with hsa_circ_0003141-shRNA1-transfected Huh-7 cells.** (**A**) Xenograft tumor volume monitored weekly. (**B**, **C**) Xenografts tumors were photographed and calculated. (**D**) Expression of UBAP2 and cleaved caspase 3 in tumor tissues analyzed by western blotting. (**E**, **F**) The relative expression of UBAP2 and cleaved caspase 3 in tumor tissues normalized to β-actin. **P < 0.01, compared with the shRNA-NC group. ^##^P < 0.01, compared with the circRNA_shRNA1 group.

To investigate the role of hsa_circ_0003141 in the regulation of HCC tumor growth *in vivo*, we established a mouse Huh-7 subcutaneous xenograft model. As shown in Figure 8A–8C, downregulation of hsa_circ_0003141 significantly inhibited the tumor volume and tumor weight, compared with shRNA-NC group. In addition, the expression of UBAP2 was significantly decreased, and the expression of cleaved caspase 3 was increased in tumor tissues following transfection with hsa_circ_0003141-shRNA1; however, this was reversed after transfection with miR-1827 inhibitor (Figure 8D–8F). These results indicate that hsa_circ_0003141 suppression inhibits the HCC tumorigenesis *in vivo*.

## DISCUSSION

CircRNAs may serve as disease-specific biomarkers for disease diagnosis [20], and play a critical role in HCC [21]. For example, circ-FOXP1 inhibits proliferation and invasion of HCC cells via sponging miR-875-3p and miR-421 [22]. The study by Lin et al has indicated that hsa_circ_0091570 acts as a sponge for miR-1307, and suppresses HCC progression [23]. However, the specific function of most circRNAs in HCC tumorigenesis is unknown.

Wang et al only identified one overlapping DEcircRNA, hsa_circ_0000517, in GSE97332 and GSE94508 datasets using Venn diagram [20]. We reanalyzed these two datasets, whereas the thresholds for the screening of DEcircRNAs we defined were different with the previous study. Using bioinformatics analysis, we have identified a novel circRNA named hsa_circ_0003141, which is significantly upregulated in human HCC tissues. Our results demonstrate that downregulation of hsa_circ_0003141 inhibits proliferation and invasion of HCC cells, and induces their apoptosis. In addition, knockdown of hsa_circ_0003141 reduces EMT in HCC cells.

Recent studies have shown that circRNAs may function as competing endogenous RNAs (ceRNAs) to recruit miRNAs, thus leading to posttranscriptional downregulation of miRNA targets [24]. The circRNA-miRNA-mRNA axis may act as a network of gene expression regulators, and the dysregulated circRNAs may contribute to HCC progression [25]. For example, a previous study by Zhan et al has indicated that hsa_circRNA_103809 promotes the progression of HCC via regulating the hsa_circRNA_103809/ miR-377-3p/FGFR1 axis [26]. In this study, we have found that miR-1827 is a potential binding target of hsa_circ_0003141 in HCC. Moreover, our data indicate that hsa_circ_0003141 functions as a ceRNA of miR-1827 in HCC, and that UBAP2 is a potential binding target of miR-1827. Yao et al indicated that overexpressed FZD4 could contribute to HCC progression, while overexpression of miR-1827 could suppress the expression of FZD4 in HCC cells, indicating that miR-1827 may display an antitumor effect in HCC [27]. We have found that miR-1827 has a complementary sequence to both hsa_circ_0003141 and UBAP2. Since downregulation of hsa_circ_0003141 reduces UBAP2 expression, but this is reversed by miR-1827 inhibitor, these data indicate that hsa_circ_0003141 promotes HCC tumorigenesis by sponging miR-1827, resulting in the increased UBAP2 expression. Feng et al have shown that circRNA ITCH inhibits HCC progression by sponging miR-214, thus upregulating linear ITCH expression; this is consistent with our results [28]. Our current data show that downregulation of hsa_circ_0003141 inhibits the progression of HCC via regulating the hsa_circ_0003141/ miR-1827/UBAP2 axis.

Ubiquitin associated protein 2 (UBAP2) participates in the regulation of cellular process including apoptosis, metastasis, and tumor growth [29]. A previous study has suggested that UBAP2 acts as a tumor suppressor in HCC, since downregulation of UBAP2 promoted proliferation and invasion of HCC cells [29]. However, our results show that high UBAP2 expression correlates with poor overall survival in patients with HCC, indicating that UBAP2 serves as an oncogene in HCC. The oncogenic role of UBAP2 is supported by the study of Latonen et al that has shown that the expression of UBAP2 is significantly upregulated in prostate tumor samples [30].

In summary, our results demonstrate that the expression of hsa_circ_0003141 is significantly increased in human HCC tissues. Downregulation of hsa_circ_0003141 inhibits proliferation, invasion, and EMT in HCC cells in vitro, and reduces tumor growth in a mouse HCC xenograft model. Our data indicate that hsa_circ_0003141 promotes the HCC progression by sponging miR-1827, thus increasing the UBAP2 expression. Therefore, targeting the hsa_circ_0003141/miR-1827/UBAP2 axis might represent a novel therapeutic option for the treatment of HCC.

## MATERIALS AND METHODS

### Data acquisition and identification of DEcircRNAs

The GSE97332 and GSE94508 datasets were downloaded from the Gene Expression Omnibus database (GEO, https://www.ncbi.nlm.nih.gov/geo/). GSE97332 dataset contains circRNAs expression data of 7 HCC tissues and 7 matched normal tissues. The GSE94508 dataset contains circRNAs expression data of 5 HCC tissues and 5 matched normal tissues. The DEcircRNAs in HCC and normal tissues were identified using R language. CircRNAs with adjusted P<0.001 and |log2 (FC)|>2 were considered as significant DEcircRNAs. The overlapping DEcircRNAs from the two datasets (GSE97332 and GSE94508) were identified using the VennDiagram package in R language.

### Function enrichment analyses

To analyze the function of the overlapping DEcircRNAs’ parental genes, Gene ontology (GO, http://www.geneontology.org/) and Kyoto Encyclopedia of Genes and Genomes (KEGG, http://www.genome.jp/kegg/) enrichment analyses were performed as previously described [18].

### Survival analysis

TCGA dataset (http://tcga-data.nci.nih.gov/tcga) was used to assess the prognostic value of the overlapping DEcircRNAs’ parental genes between HCC tissues and matched normal tissues; P<0.05 was considered statistically significant.

### RT-qPCR

Total RNA was extracted from HCC cells using TRIzol (Thermo Fisher Scientific, Waltham, MA, USA). EntiLink™ 1^st^ Strand cDNA Synthesis Kit (ELK Biotechnology, Hubei, China) was used to synthesize cDNA. The specific primer for miR-1827 was as follows: 5’-CTCAACTGGTGTCGTGGAGTCGGCAATTCAGTTGAGATTCAATC-3’. The specific primer for U6 was as follows: 5’-AACGCTTCACGAATTTGCGT-3’. Real-time PCR was performed using SYBR Select Master Mix (Applied Biosystems, Foster City, CA, USA) on Applied Biosystems 7300 Real-Time PCR System. The PCR primers were as follows: miR-1827, forward, 5’-GGTGAGGCAGTAGATTGAATCTC-3’; reverse, 5’-CTCAACTGGTGTCGTGGAGTC-3’. U6, forward, 5’-CTCGCTTCGGCAGCACAT-3’; reverse, 5’-AACGCTTCACGAATTTGCGT-3’. Actin, forward, 5’-GTCCACCGCAAATGCTTCTA-3’; reverse, 5’-TGCTGTCACCTTCACCGTTC-3’. Hsa_circ_0003141, forward, 5’-AGAGCCTGGATTTGGACGTG-3’; reverse, 5’-TCTGCACCATTCTGAGCAGC-3’. Actin and U6 were used as internal controls for circRNA and miRNA, respectively, and the hsa_circ_0003141 and miR-1827 levels were calculated using the 2^-ΔΔCt^ method.

### Cell culture

Human hepatoma cell line Huh7 was purchased from Procell (Wuhan, China). Human hepatoma cell line Hep3B2.1-7 was purchased from Beyotime Biotechnology (Beijing, China). Human hepatocellular carcinoma cell line HepG2 was purchased from American Type Culture Collection (ATCC, Rockville, MD, USA). Human hepatocarcinoma cell line HCCLM3 was obtained from the China Center for Type Culture Collection (CCTCC, Wuhan, China). Cells were maintained in Dulbecco’s modified Eagle’s medium (DMEM, Thermo Fisher Scientific) with 10% fetal bovine serum (FBS, Hyclone, Logan, UT, USA) at 37°C in a 5% CO_2_ humidified incubator.

### Lentivirus production and cell transfection

Two pairs of cDNA oligonucleotides inhibiting hsa_circ_0003141 expression were inserted into the pLVX-IRES-PURO vector (GenePharma, Shanghai, China), hsa_circ_0003141-shRNA1 (5’-CCGGATGTGAACAAAGCTATCAATACTCGACTTTGTTCACATTTTTTG-3’) and hsa_circ_0003141-shRNA2 (5’-CCGGCCACAGCCCAAACACATCAATTGATGTGTTTGGGCTGTGGTTTTT-3’), which were then hsa_circ_0003141 transfected into 293T cells. A control scrambled shRNA sequence was specifically designed and inserted into the pLVX-IRES-PURO vector, which was used as a negative control (NC). The shRNA-NC plasmids were then transfected into 293T cells. After 72 h incubation, cell supernatants were collected, and used for transfection (24 h) of Huh-7 and Hep3B2.1-7 cells with shRNA-NC, hsa_circ_0003141-shRNA1 and hsa_circ_0003141-shRNA2, respectively. Cells were then cultured (48 h) with 2.5 μg/mL puromycin (Thermo Fisher Scientific) to select stable hsa_circ_0003141 knockdown cells. Cells of the blank control group were not transfected.

MiR-1827 mimics, miR-1827 inhibitor and NC, obtained from GenePharma (Shanghai, China), were transfected into Huh-7 and Hep3B2.1-7 cells using Lipofectamine 2000 (Thermo Fisher Scientific) respectively. The culture medium containing 10% FBS was changed 6 h after transfection, and the cells were incubated for another 48 h at 37°C. Cells of the blank control group were not transfected.

### Cell proliferation assay

Huh-7 and Hep3B2.1-7 cells (5000 cells/well) were seed onto 96-well plates and incubated overnight at 37 °C. Cells were then transfected with hsa_circ_0003141-shRNA1 and shRNA-NC for 0, 24, 48, and 72 h, and cell proliferation was measured using the Cell Counting Kit-8 (CCK8, Beyotime, Shanghai, China) according to the manufacturer's protocol. Absorbance was measured at a wavelength of 450 nm using a microplate reader (Bio-Rad Laboratories, Inc., Hercules, CA, USA).

### Cell apoptosis assay

Huh-7 and Hep3B2.1-7 cells were washed with cold PBS, and then incubated in 75% ethanol at -20°C overnight. Cells were stained with 5 μL of propidium iodide (PI) and Annexin V-FITC (Thermo Fisher Scientific) for 20 min in dark at room temperature (RT), and apoptotic cells were analyzed by a flow cytometer (FACScan™, BD Biosciences, Franklin Lakes, NJ, USA).

### Transwell invasion assay

Transwell invasion assay was performed using 24-well transwell chambers (0.8 μm; Corning New York, NY, USA) with Matrigel coating. Huh-7 cells (4 x 10^4^) were suspended in 200 μL of serum-free DMEM, and seeded onto the upper chamber. After that, 600 μL of DMEM medium supplemented with 10% FBS was added into the lower chamber. Cells that invaded through the membrane were fixed with 4% paraformaldehyde, stained with 0.2% crystal violet, and photographed using a laser confocal microscope (Olympus CX23 Tokyo, Japan).

### Western blot assay

Huh-7 cells were lysed using RIPA buffer, and the protein concentration was quantified using BCA method (Beyotime Institute of Biotechnology). Proteins were separated by 10% sodium dodecyl sulfate-polyacrylamide gel electrophoresis, and transferred onto PVDF membrane (Thermo Fisher Scientific). After blocking in 5% skimmed milk in TBST (1 h, RT), the membranes were incubated (4°C, overnight) with the following primary antibodies: anti-E-cadherin (1:1000, Abcam), anti-N-cadherin (1:1000, Abcam), anti-α-SMA (1:1000, Abcam), anti-UBAP2 (1:1000, Abcam), anti-cleaved caspase 3 (1:1000, Abcam), and anti-β-actin (1:1000, Abcam). After washing, the membranes were incubated (1 h, RT) with secondary antibody (Abcam, 1: 5000), and visualized using electrochemiluminescence (Thermo Fisher Scientific). β-actin was used as an internal control.

### Dual-luciferase reporter assay

The binding sequences of miR-1827 in hsa_circ_0003141 were cloned into psiCHECK-2 vector (Applied Biosystems, USA). After that, cells were co-transfected with WT-hsa_circ_0003141 or MT-hsa_circ_0003141 plasmid, with miR-1827 mimics respectively using Lipofectamine 2000 (Invitrogen, Carlsbad, CA, USA). Moreover, cells were co-transfected with WT-hsa_circ_0003141 or MT-hsa_circ_0003141 plasmid, with mimics NC using Lipofectamine 2000, which was considered as the vector-control group. Meanwhile, cells were transfected with WT-hsa_circ_0003141 or MT-hsa_circ_0003141 plasmid, which was considered as the control group. For UBAP2 reporter assay, UBAP2 segment was synthesized with either wild-type (WT) or mutant (MT) seed region and cloned into the psiCHECK-2 vector. Cells were then co-transfected with WT-UBAP2 or MT-UBAP2 plasmid, with miR-1827 mimics or mimics NC respectively using Lipofectamine 2000. After 48 h, the Dual Luciferase Reporter Assay System (Promega, Madison, USA) was used to detect luciferase activity with renilla luciferase activity as endogenous control.

### Fluorescence in situ hybridization analysis

The oligonucleotide probe for hsa_circ_0003141 was synthesized by Biosense (Guangzhou, China). Huh-7 cells were incubated with 10 μL of the probe mixture (Biosense) at 37°C in the dark overnight. Cell nuclei were stained 20 min with 4,6-diamidino-2-phenylindole (DAPI; Thermo Fisher Scientific) for 20 min, and visualized using a fluorescence microscope (Olympus) as previously described [19].

### Animal study

BALB/c nude mice (n = 18, 6 – 8 weeks old) were obtained from the Charles River (Beijing, China). Animals were randomized into three groups: blank, shRNA-NC, and hsa_circ_0003141-shRNA1 group. 5 x 10^6^ Huh-7 or Huh-7 cells stably expressing shRNA-NC, hsa_circ_0003141-shRNA1 cells in 100 μL PBS were injected into the left flank of nude mice. The tumor volume was calculated by the following formula: V = (length x width^2^)/2. After 5 weeks, animals were sacrificed under anesthesia by following the recommended procedures of the National Institutes of Health guide for the care and use of laboratory animals. All animal experiments were approved by the Institutional Animal Care and Use Committee at the Union Hospital, Tongji Medical College, Huazhong University of Science and Technology.

### Statistical analysis

All experiments were repeated three times. Data are presented as mean ± standard deviation (S. D.). Graphs were generated using GraphPad Prism software (version 7.0, La Jolla, CA, USA). One-way analysis of variance (ANOVA) and Tukey’s tests were carried out for multiple group comparisons. A P-value < 0.05 was considered as statistically significant.

## Supplementary Material

Supplementary Figures
