# Supplementary material for: Circular RNA hsa_circ_0003141 promotes tumorigenesis of hepatocellular carcinoma via a miR-1827/UBAP2 axis
Source: Aging (Albany NY). 2020 May 28;12(10):9793–806. doi: 10.18632/aging.103244 (PMC7288939; doi:10.18632/aging.103244)
Supplement: Supplementary Figures [file aging-12-103244-s001..pdf]

SUPPLEMENTARY FIGURES

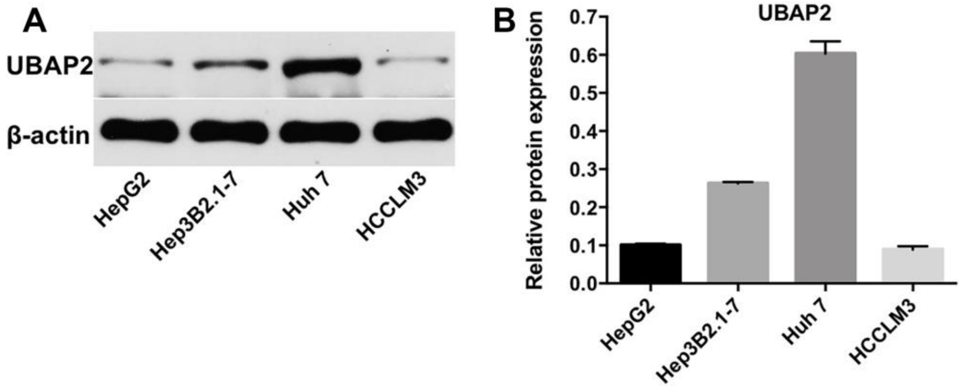

Supplementary Figure 1. Relative UBAP2 expression in HCC cells. (**A**, **B**) Western analysis of UBAP2 in HepG2, Hep3B2.1-7, Huh-7 and HCCLM3 cells. The relative expression of UBAP2 in HepG2, Hep3B2.1-7, Huh-7 and HCCLM3 cells normalized to β-actin.

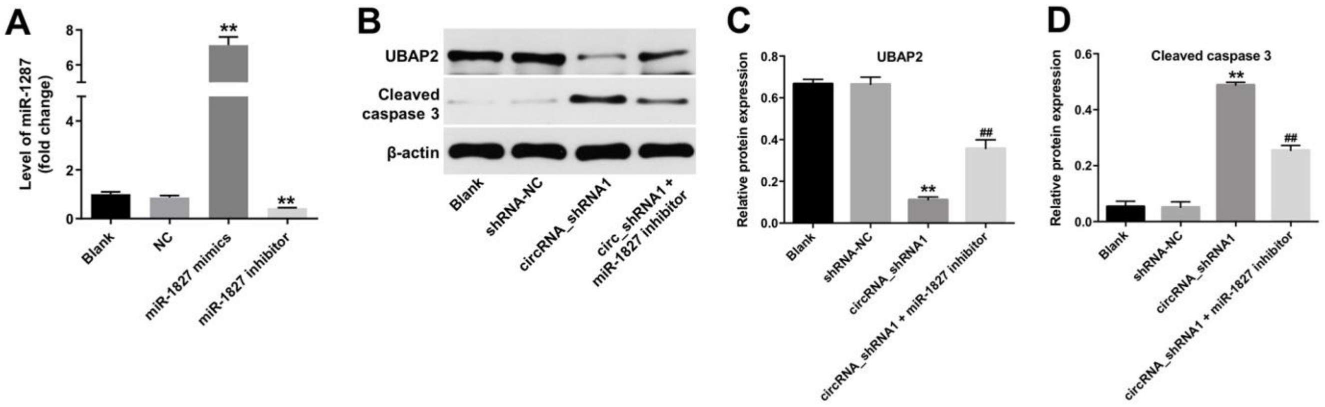

Supplementary Figure 2. Hsa_circ_0003141 functions as oncogene in Hep3B2.1-7 cells by sponging miR-1827, and thus increasing UBAP2 expression. (**A**) The level of miR-1827 in Hep3B2.1-7 cells transfected with miR-1827 mimics or miR-1827 inhibitor analyzed by qRT-PCR. **P < 0.01, compared with the NC group. (**B**) Western analysis of UBAP2 and cleaved caspase 3 in Hep3B2.1-7 cells transfected with hsa_circ_0003141-shRNA1 with and without miR-1827 inhibitor. (**C**, **D**) The relative expression of UBAP2 and cleaved caspase 3 in Hep3B2.1-7 cells normalized to β-actin. **P < 0.01, compared with the shRNA-NC group. ##P < 0.01, compared with the circRNA_shRNA1 group.
